# Comparison of the standard WHO susceptibility tests and the CDC bottle bioassay for the determination of insecticide susceptibility in malaria vectors and their correlation with biochemical and molecular biology assays in Benin, West Africa

**DOI:** 10.1186/1756-3305-6-147

**Published:** 2013-05-20

**Authors:** Nazaire Aïzoun, Razaki Ossè, Roseric Azondekon, Roland Alia, Olivier Oussou, Virgile Gnanguenon, Rock Aikpon, Gil Germain Padonou, Martin Akogbéto

**Affiliations:** 1 Centre de Recherche Entomologique de Cotonou (CREC), Cotonou, 06 BP: 2604, Bénin; 2 Faculté des Sciences et Techniques de l’Université d’Abomey Calavi, Calavi, Bénin

**Keywords:** Susceptibility, Insecticide, WHO bioassay, CDC bioassay, Synergist, *Anopheles gambiae*

## Abstract

**Background:**

The detection of insecticide resistance in natural populations of *Anopheles* vectors is absolutely necessary for malaria control. In the African region, the WHO insecticide susceptibility test is the most common method for assessing resistance status. In order to search for a simple, rapid and more reliable technique in the assessment of insecticide resistance in malaria vectors, we compared the WHO tests with the CDC bottle bioassay in the Ouemé province of southern Benin where insecticide resistance has been widely reported.

**Methods:**

Larvae and pupae of *Anopheles gambiae s.l.* mosquitoes were collected from the breeding sites in Ouemé. WHO and CDC susceptibility tests were conducted simultaneously on unfed female mosquitoes aged 2–5 days old. WHO bioassays were performed with impregnated papers of deltamethrin (0.05%) and bendiocarb (0.1%), whereas CDC bioassays were performed with stock solutions of deltamethrin (12.5 μg per bottle) and bendiocarb (12.5 μg per bottle). PCR techniques were used to detect species, *Kdr* and *Ace-1* mutations. CDC biochemical assays using synergists were also conducted to assess the metabolic resistance.

**Results:**

A slight decrease in mortality rates was observed with 97.95% and 98.33% obtained from CDC and WHO bioassays respectively in populations of mosquitoes from Adjara and Dangbo. PCR revealed that all specimens tested were *Anopheles gambiae s.s.* The *Kdr* mutation was found at high frequency in all populations and both the *Kdr* mutation and mono-oxygenase enzymes were implicated as mechanisms of pyrethroid resistance in *An. gambiae* from Misserete.

**Conclusion:**

This study emphasizes that both WHO and CDC bioassays give similar results with regards to the susceptibility of mosquitoes to insecticides in southern Benin. There were complementarities between both methods, however, some specificity was noted for each of the two methods used. Both *Kdr* and metabolic mechanisms were implicated in the resistance.

## Background

Vector resistance to pyrethroids has been reported in many African countries, including West Africa (Ivory Coast, Burkina Faso, Benin, Senegal) [1-6], central Africa (Cameroon) [4,7], East Africa (Kenya) [8] and Southern Africa (South Africa) [9]. The development of resistance is a complex and dynamic process and depends upon many factors. Increasing the dosages of insecticide in an attempt to maintain efficacy is not a recommended option because of environmental and safety concerns. The resistance genes in the vector population may also be driven to even higher frequencies. Most commonly, when the frequency of resistant insects in a vector population increases, efficacy of the treatment decreases up to the point where the insecticide has to be replaced by another one. Therefore, the management of insecticide resistance is a major issue, which must interest the different National Malaria Control Programmes. This management requires two kinds of information: sound knowledge of the mechanisms of resistance and a thorough resistance monitoring programme. The characterization of involved resistance mechanisms allows us to appreciate and predict their impact on vector control strategies. Routine monitoring of insecticide resistance in the natural populations of vectors helps us to detect early resistance and improve effectiveness of operational control strategies.

Currently, insecticide resistance in mosquito populations is detected in Africa following WHO guidelines [10]. According to this protocol, mosquito samples are exposed to a series of different insecticides using insecticide-impregnated papers with a single discriminating or diagnostic dose for each insecticide. This method has been widely used in the field and gives satisfactory results in detecting insecticide resistance for surveillance purposes. After the exposure of mosquitoes to insecticide impregnated papers, they are held in the absence of insecticide for 24 hours before mortality is recorded.

The WHO resistance test kits are expensive and test papers are not available for some insecticides, such as dibrom or resmethrin, being restricted to those insecticides that are approved by WHO for routine use in vector control [10,11]. The insecticide diagnostic dosages available are not applicable to all vector species. No provision is made in the WHO test kit for using synergists to evaluate potential biochemical resistance mechanisms [10,11]. In rural areas, it is difficult to meet all conditions that the WHO method requires and entomologists working in peripheral areas carrying out routine surveillance for the National Malaria Control Programmes (NMCP) need a technique that is simple, rapid, economical and practicable in the field.

Another approach for detection of resistance, the CDC bottle bioassay, is rarely used in Africa. The method utilizes 250 ml glass Wheaton bottles treated with an insecticide [11]. The advantages and disadvantages of this technique compared with the WHO method are given in Table 1. One of the advantages is that synergists can be added at the same time as the insecticide, in case of resistance, to check for the existence of biochemical resistance mechanisms [11]. We know that both techniques do not have the same principles. Nevertheless there is a need to compare them in order to check if they converge on similar results. In addition, it is also important to check both methods for their specificities and complementarity.

**Table 1:** Comparison of advantages and drawbacks of both WHO and CDC methods

		
**Advantages**	**WHO**	-WHO papers are always ordered in the impregnated form
		-Knock down (Kd) or dead mosquitoes recording in WHO tubes is easy
		-insecticide diagnostic doses recommended by WHO for susceptibility tests are standard
		-WHO assay requires the purchase of all components (WHO kit) from a centralized source and that allows easy comparison of results from one year to another and from one
		study site to another
	**CDC**	-CDC bioassay uses less mosquitoes than WHO bioassay
		-CDC bottles bioassay does not need mosquitoes transferred from one bottle to another
		-CDC bioassay allows detection of simple or multiple resistance mechanisms in insecticide resistant mosquitoes
		-bottle assay is simple and rapid
		-some of the components of bottle assay (CDC kit) are more readily and cheaply available
		-any concentration of any insecticide (pure or formulated) may be evaluated with bottle assays
		-bottle bioassay can also measure the efficacy of an insecticide formulation
**Drawbacks**	**WHO**	-mosquitoes transferred from one tube to another need care during WHO cylinder tube test
		-WHO bioassay requires 24 hours mortality recording after putting mosquitoes in stable conditions of temperature and humidity
		-no provision is made in WHO kit for using synergist in detection of metabolic resistance mechanisms
		-increasing the cost of WHO kit and logical complexity of the assay
	**CDC**	-CDC bottles need to be coated with insecticide by oneself before each bioassay
		-shelf-life and re-use of pre-prepared bottles are still not well documented or studied in laboratory conditions
		-mortality recording in Wheaton bottles necessitates care and is not easy

The goal of this study, therefore, was to compare both methods and show the limits of the CDC method and its real advantages with regard to the WHO method.

## Methods

### Study area

**Figure 1:**
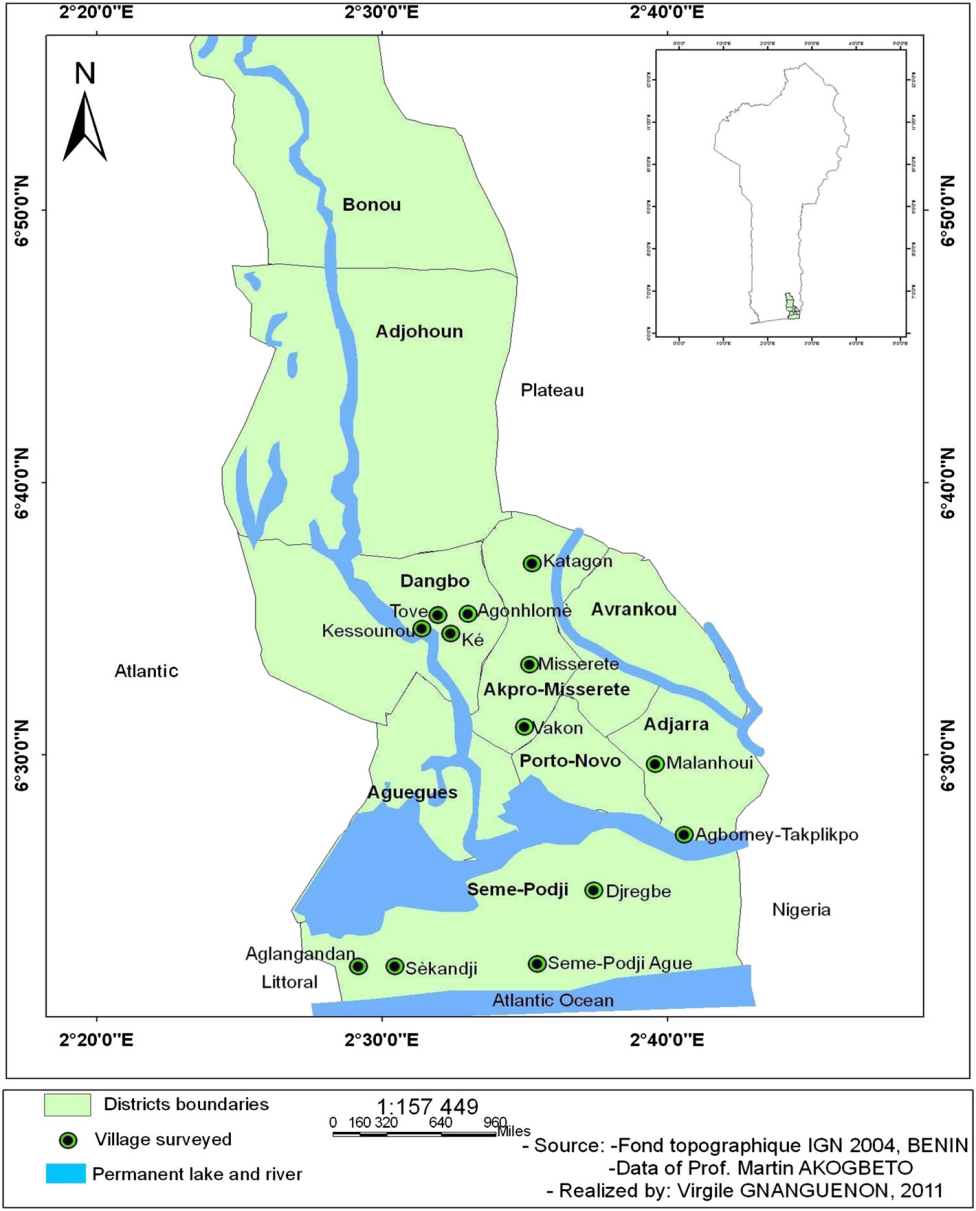
Map of the study area.

The study was carried out in the south of Benin in Dangbo, Misserete, Seme and Adjara districts of Ouemé province (Figure 1). The choice of the study sites took into account the economic activities of populations, their usual protection practices against mosquito bites, the indoor residual spraying (IRS) in progress in these localities and peasant practices to control farming pests. These factors have an impact on resistance development in the local vector mosquitoes. We took them into account to compare both methods according to the resistance level. Ouemé has a climate with four seasons, two rainy seasons (March-July and September-November) and two dry seasons (December-March and August-September). The temperature ranges from 25 to 30°C with the annual mean rainfall between 900 and 1500 mm.

### Mosquito collection

*Anopheles gambiae s.l.* mosquitoes were collected during the rainy seasons (March-July and September-November 2010) across the four districts selected in south Benin. Larvae and pupae were collected from breeding sites and kept in separate labeled bottles related to each locality. The samples were reared to adults in the CREC (Centre de Recherche Entomologique de Cotonou, Benin) insectary. *Anopheles gambiae* Kisumu, a reference susceptible strain, was used as a control for the bioassay tests.

Susceptibility tests were done simultaneously following WHO and CDC protocols on unfed female mosquitoes aged 2–5 days old, reared from the larval and pupal collections. Each *An. gambiae s.l.* sample was separated into two batches: batch 1 was used for susceptibility tests following the WHO protocol and batch 2 for CDC susceptibility tests. All susceptibility tests were conducted in the CREC laboratory at 25+/−2°C and 70 to 80% relative humidity.

### WHO protocol

The principle of the WHO bioassay is to expose insects to a given dose of insecticide for a given time to assess susceptibility or resistance.

The standard WHO discriminating dosages are twice the experimentally derived 100% lethal concentration (LC100 value) of a reference susceptible strain [12]. In this study, two insecticides were tested: deltamethrin (0.05%) and bendiocarb (0.1%). The choice of bendiocarb was justified by its use for Indoor Residual Spraying (IRS) in Ouemé, whereas deltamethrin is the insecticide used on PermaNets that are distributed free by the NMCP in the swampy areas of Ouemé.

An aspirator was used to introduce 20 to 25 unfed female mosquitoes aged 2–5 days from batch 1 into five WHO holding tubes (four tests and one control) that contained untreated papers. They were then gently blown into the exposure tubes containing the insecticide-impregnated papers. After one-hour exposure, mosquitoes were transferred back into holding tubes and provided with cotton wool moistened with a 10% honey solution. The number of mosquitoes “knocked down” at 60 minutes and mortalities at 24 hours were recorded following the WHO protocol [12].

### CDC protocol

The principle of CDC bottle bioassay is to determine the time it takes an insecticide to penetrate an arthropod, traverse its intervening tissues, get to the target site, and act on that site relative to a susceptible control. Anything that prevents or delays the compound from achieving its objective of killing the arthropods contributes to resistance.

Diagnostic doses that were applied in the present study were the doses recommended by CDC [13]. These doses were checked on the *An. gambiae* Kisumu susceptible reference strain before being applied to field populations. For *An. gambiae s.l.*, the diagnostic dose of 12.5 μg per bottle for both deltamethrin and bendiocarb was used for a diagnostic exposure time of 30 minutes.

The solutions were prepared and the bottles coated according to the CDC protocol [13].

Fifteen to 20 unfed female mosquitoes aged 2–5 days from batch 2 were introduced into four 250 ml Wheaton bottles coated with insecticide and one control bottle coated with acetone only. The number of dead or alive mosquitoes was monitored at different time intervals (15, 30, 35, 40, 45, 60, 75, 90, 105, 120 minutes). This allowed us to determine the total percent mortality (Y axis) against time (X axis) for all replicates using a linear scale.

### Biochemical assays using synergists

**Figure 2:**
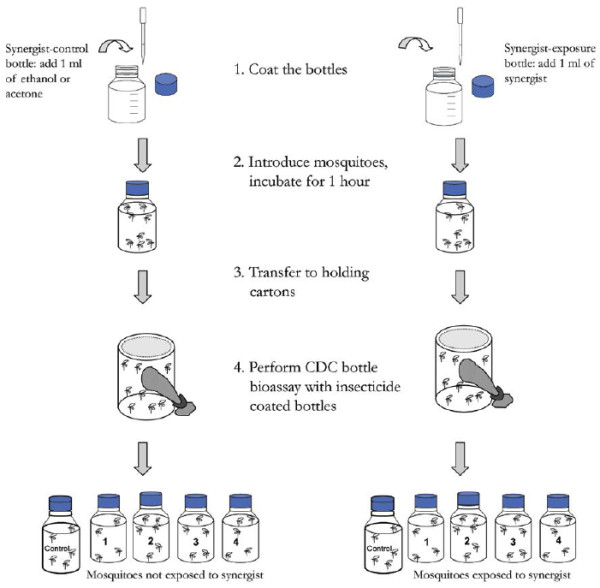
**Diagram for performing the CDC bottle bioassay with synergists**[13]**.**

Synergists were used according to the protocol described by CDC [11,13] following the procedure outlined in Figure 2. Samples that showed high resistance to pyrethroids from Misserete district were exposed to the effects of two synergists: S.S.S-tributylphosphorotrithioate (DEF) (125 μg/bottle), which inhibits esterase activity; and piperonyl butoxide (PBO) (400 μg/bottle), which inhibits oxidase activity. These two synergists were used separately and in combination.

Approximately 125 mosquitoes were used for each synergist assay. The number of dead or alive mosquitoes was monitored at different time intervals (0, 15, 30, 35, 40, 45, 60, 75, 90, 105, 120 minutes). This test allowed us to compare the obtained percentages of dead mosquitoes (Y axis) against time (X axis) before the addition of the synergist (s) to those obtained after the addition of the synergist (s) (Figure 2).

### PCR detection of species and the *Kdr* and *Ace-1* mutations

At the end of WHO and CDC bioassays, polymerase chain reaction tests for species identification [14] was performed to identify the members of *An. gambiae* complex collected from each site. PCR for the detection of the *Kdr* “Leu-phe” mutation was carried out on dead and alive *An. gambiae* mosquitoes as described by Martinez-Torres *et al.*[15]. The PCR-RFLP diagnostic test was used to detect the presence of the G119S mutation (*Ace-1* gene) as described by Weill *et al.*[16].

### Statistical analysis and data interpretation

The resistance status of mosquito samples from batch 1 was determined according to the latest WHO criteria [10] as follows:

– Mortality rates between 98%-100% indicate full susceptibility

– Mortality rates between 90%-97% require further investigation

– Mortality rates < 90%, the population is considered resistant to the tested insecticides.

The resistance status of mosquito samples from batch 2 was determined according to the CDC criteria [11,13]. The susceptibility thresholds at the diagnostic time of 30 minutes for both pyrethroids and carbamates are:

– Mortality rate = 100%: the population is fully susceptible

– Mortality rate < 100%: the population is considered resistant to the tested insecticides.

Abbott’s formula was not used in this study for the correction of mortality rates in either the test-tubes or test-bottles because the mortality rates in all controls was always less than 5% [17].

Molecular results (*Kdr* and *Ace-1* frequencies) were correlated with the results of insecticide susceptibility tests performed with both WHO and CDC methods from each of the districts surveyed.

Analysis using Fisher’s exact test and test of proportion was performed on the data sets gathered from the localities surveyed to compare each of two tested insecticides and assess the resistance status of each tested *An. gambiae* population using both WHO and CDC methods. To appreciate the effects of the synergists PBO and DEF on *An. gambiae* Misserete populations resistant to deltamethrin, we used Kruskal-Wallis test. The significance level was set at 5%.

## Results

### Mosquito species identification

PCR revealed 100% of mosquitoes tested were *Anopheles gambiae s.s*. (Tables 2 and 3).

**Table 2:** ***Kdr *frequency in surviving *An. gambiae *populations 24 h post-exposure to deltamethrin**

			***Kdr *mutation**				***Ace-1 *mutation**			
**Locality**	**Number of survivors tested**	**Species Ag**	**RR**	**RS**	**SS**	**F( *Kdr *)**	**RR**	**RS**	**SS**	**F( *Ace-1 *)**
Adjara	27	27	19	7	1	0.83	0	0	27	0
Dangbo	25	25	19	6	0	0.88	0	0	25	0
Misserete	22	22	18	4	0	0.91	0	0	22	0
Seme	25	25	20	5	0	0.90	0	0	25	0

Ag: *An. gambiae s.s.*

**Table 3:** ***Kdr *frequency in dead *An. gambiae *populations 24 h post-exposure to deltamethrin and bendiocarb**

			***Kdr *mutation**				***Ace-1 *mutation**			
**Locality**	**Number of dead tested**	**Species Ag**	**RR**	**RS**	**SS**	**F( *Kdr *)**	**RR**	**RS**	**SS**	**F( *Ace-1 *)**
Adjara	27	27	19	7	1	0.83	0	0	27	0
Dangbo	25	25	15	6	4	0.72	0	0	25	0
Misserete	22	22	18	4	0	0.91	0	0	22	0
Seme	25	25	19	4	2	0.84	0	0	25	0

Ag: *An. gambiae s.s.*

### Susceptibility of *An. gambiae* populations to pyrethroids and carbamates

**Figure 3:**
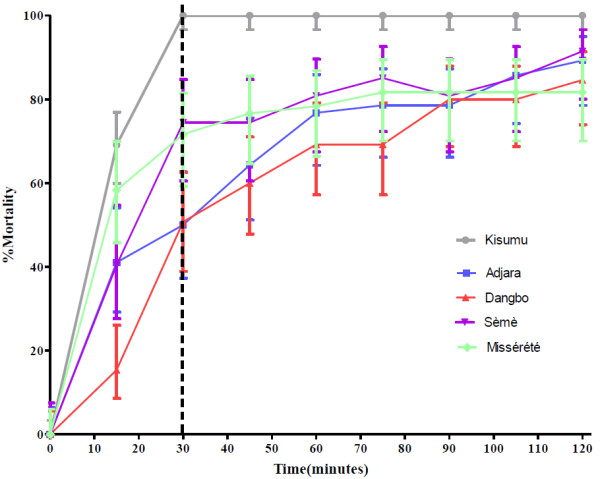
**Mortality of *An. gambiae *Kisumu, Adjara, Dangbo, Misserete and Seme populations observed after two hours exposure to CDC bottles treated with deltamethrin (1.25%).**

**Figure 4:**
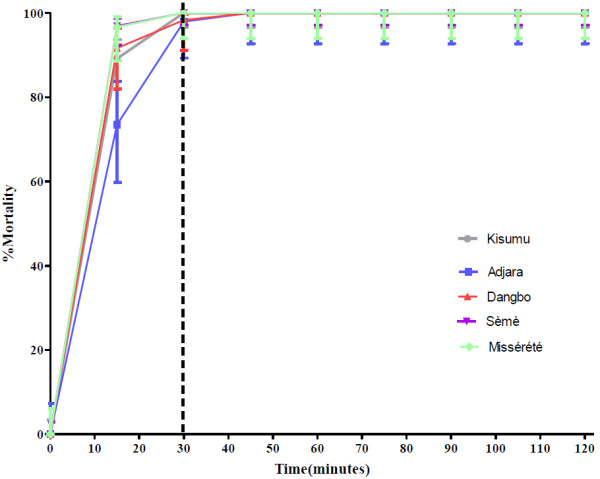
**Mortality of *An. gambiae *Kisumu, Adjara, Dangbo, Misserete and Seme populations observed after two hours exposure to CDC bottles treated with bendiocarb (1.25%).**

The results of 24 hours mortality recorded after exposure of mosquitoes to impregnated papers of deltamethrin and bendiocarb were compared to those recorded from CDC bottle bioassays at the susceptibility threshold of 30 minutes. The Kisumu strain (control) confirmed its susceptibility status as a reference strain with 100% mortality after exposure to deltamethrin and bendiocarb following both methods (Table 4, Figures 3 and 4). All the *An. gambiae* populations were resistant to deltamethrin according to both methods (Table 4, Figure 3) and susceptible to bendiocarb (Table 4, Figure 4), although one mosquito from each of the three localities, Adjara, Dangbo and Misserete, survived exposure to the carbamate. The *An. gambiae* Seme population did not show full resistance to deltamethrin according to WHO 1998 criteria [12] and so tests were repeated giving similar results. Under the new 2013 WHO criteria [10] this population is classified as resistant. Table 4 shows that the WHO and CDC methods gave comparable results. There was no significant difference between mortality rates of *An. gambiae* Seme populations to deltamethrin recorded from both methods (p = 0.2001).

**Table 4:** Susceptibility data recorded according to both WHO and CDC methods

		**Number tested**		**% Kd at 60 min**	**% Mortality**		**Resistance status**	
Populations	Insecticides	WHO	CDC	WHO	WHO	CDC	WHO	CDC
Kisumu (control)	Deltamethrin	103	110	100	100	100	S	S
	Bendiocarb	99	111	100	100	100	S	S
Adjara	Deltamethrin	51	56	60.78	49.01	50	R	R
	Bendiocarb	40	49	100	100	97.95	S	S
Dangbo	Deltamethrin	84	65	67.85	73.8	50.76	R	R
	Bendiocarb	45	60	100	100	98.33	S	S
Misserete	Deltamethrin	54	60	66.66	70.37	71.66	R	R
	Bendiocarb	100	60	100	99	100	S	S
Seme (without repetition of bioassays)	Deltamethrin	99	47	77.77	84.84	74.46	**R**	**R**
	Bendiocarb	49	131	100	100	100	S	S
Seme (with repetition of bioassays)	Deltamethrin	49	27	55.10	89.79	74.07	**R**	**R**
	Bendiocarb	49	131	100	100	100	S	S

S, Susceptible; R, Resistant.

### Multiple insecticide resistance mechanisms in *Anopheles gambiae* to pyrethroids

**Figure 5:**
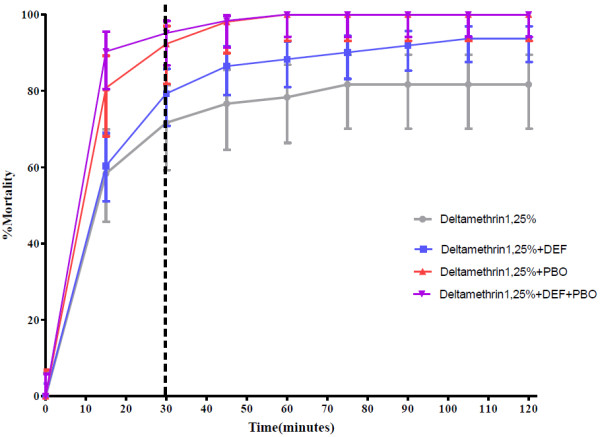
**Implication of mono-oxygenases in resistance of *An. gambiae *to pyrethroids in Misserete district.**

The data presented in Figure 5 show that after exposure to the synergists PBO and DEF prior to exposure to deltamethrin 1.25%, the percentage of dead mosquitoes from Misserete on PBO was higher than that obtained with deltamethrin alone. The PBO synergist did not eliminate deltamethrin resistance, but significantly reduced the level with the mortality rate increasing from 71.66% to 92.30%. The DEF synergist, on the other hand, had no effect, with 20% of mosquitoes continuing to fly after 30 minutes (susceptibility threshold) and more than 5% flying after 2 hours. The use of the synergist combination DEF + PBO gave the same result as the one obtained with PBO alone (p > 0.05). These results suggest an implication of mono-oxygenases in resistance of *An. gambiae* to pyrethroids.

In addition to biochemical resistance noted within *An. gambiae* Misserete, the results of molecular tests performed on all populations revealed very high frequencies of the *Kdr* mutation ranging between 0.80 and 0.91 (Tables 2 and 3). The highest *Kdr* frequency (91%) was found in the *An. gambiae* Misserete population.

## Discussion

Although WHO susceptibility tests require more mosquitoes (4 test tubes containing 20 to 25 mosquitoes each plus one control tube) than those of CDC technique (4 test bottles containing 15 to 20 mosquitoes each plus one control bottle), the concordance between the results obtained with both methods is clear.

Bottle bioassays confirmed the insecticide resistance status of mosquitoes when the old WHO criteria [12] classified them as a suspicion of resistance requiring further investigation. This discrepancy fell away when the new 2013 WHO criteria [10] were used.

When the WHO susceptibility kit is not readily available, bottle bioassays can be used to determine insecticide resistance status of mosquito populations. WHO bioassays utilize cylinder plastic tubes whereas CDC bottles bioassays use 250 ml Wheaton bottles which are made from glass. WHO papers do not need to be treated by oneself before their utilization because they are ordered in the impregnated form. Conversely, CDC bottles need to be coated with insecticide by oneself before each bioassay. In fact, the shelf-life and re-use of pre-prepared bottles are still not well documented or studied in laboratory conditions. But, in field conditions, the studies of Perea *et al.*[18] showed that bottles treated with 10 μg a.i deltamethrin per bottle could be stored for at least 14 days and re-used on three occasions. Mosquitoes transferred from one tube to another requires care during WHO susceptibility tests, whereas CDC bottles bioassays do not need mosquitoes to be transferred from the exposure bottle. The recording of knock down (Kd) of mosquitoes in WHO tubes is easy, whereas the mortality recording in 250 ml Wheaton bottles necessitates care because this recording is often done by raising these bottles and putting them on their side, and that often provokes an increase in mosquitoes moving during CDC bottle bioassays. The duration of WHO tests is one hour with an obligation of 24 hours mortality recording after exposure of mosquitoes to insecticide-treated papers and requires keeping mosquitoes in stable conditions of temperature and relative humidity. The CDC test duration is two hours without deferred mortality recording. However, before performing CDC bioassays, bottle cleaning, coating and the drying of coated bottles take a long time. According to the CDC method, any concentration of any insecticide (pure or formulated) may be evaluated.

The assessment of the diagnostic dosage and diagnostic time for each insecticide used against malaria vectors, in each region and for each of the main vector species is absolutely necessary in the monitoring of insecticide resistance in vectors according to CDC procedures. However, in Africa, there are no pre-established diagnostic dosages and times for numerous insecticides used in public health against malaria vectors using the CDC protocol. There is a need to set up this method in the African region where resistance in malaria vectors is increasingly spreading. The insecticide diagnostic doses recommended by WHOPES for susceptibility tests are standard.

The slight decrease of susceptibility obtained with *Anopheles gambiae* populations exposed to bendiocarb is not synonymous with resistance. Only one mosquito from each of three localities survived exposure to the WHO test (Misserete) or the CDC test (Adjara and Dangbo). These populations will need to be monitored, however, considering the use of bendiocarb for public health purposes in southern Benin.

The apparent discordance between WHO and CDC bioassay results on the *An. gambiae* Seme population falls away when the new WHO criteria [10] for resistance are used. However, one drawback of the CDC bottle bioassay is that a proportion of mosquitoes do not have the minimum contact with the insecticide product because of the test shortness (mortality recorded at susceptibility threshold of 30 minutes) and the repellency effect of deltamethrin. This could result in an over-estimation of resistance using the CDC method. Similar results were reported elsewhere. For instance, *Anopheles nuneztovari* populations in Colombia were susceptible to fenitrothion by WHO bioassay, but using CDC methods showed 20% survival [19]. This, however, could be due the WHO assay requiring a 2-hour exposure on fenitrothion treated papers, indicating that the 30 min threshold for the CDC assay is far too short for this insecticide. Other contradictions from the same study were observed with the pyrethroids and DDT but no interpretation of these contradictory results was put forward by these authors [19].

No provision is made in the WHO assay for using synergists to evaluate potential biochemical resistance mechanisms. If synergist assays are to be performed using the WHO system then the papers must be prepared by oneself. In this respect, the CDC bottle bioassay is easier to use for synergist assays [11]. The suggestion that mono-oxygenases are implicated in resistance of *An. gambiae* Misserete populations to deltamethrin needs to be confirmed by microplate-based biochemical assays or more advanced molecular assays. Biochemical assays utilized to detect elevated oxidase and esterase activity within insects does not always equate with the phenotypic expression of resistance in WHO tests or bottle bioassays [20]. This may mean that biochemical tests are unable to correlate fully with phenotypic resistance or serve as a reliable indicator of metabolic resistance.

Our results showed that *An. gambiae* Misserete populations have developed more than one resistance mechanism to deltamethrin. In southern Benin, Corbel *et al.*[21] have already reported on multiple insecticide resistance mechanisms in *An. gambiae* from Ladji using the method described by Hemingway [22]. Among these mechanisms, mixed function oxidase (MFO) and α-esterase with the presence of *Kdr* at high frequency (80%) have been reported.

## Conclusion

This study emphasizes that both WHO and CDC bioassays give similar results with regard to mosquito susceptibility to insecticides. There is a complementarity between both methods, however, some specificities were noted for each. Molecular assays showed the presence of high frequencies of the *Kdr* mutation in all samples while synergist assays implicated mono-oxygenases in populations from one locality.

## Competing interests

The authors declare that there is no competing interest.

## Authors’ contributions

MA and NA conceived the study. GGP, RO, RAI have participated in the design of the study. Entomologic data was collected by NA, OO, RAZ, RAL, RO and bioassays and laboratory analysis were carried out by NA, RAZ and RAL. MA and NA have participated in the analysis and interpretation of data. VG has contributed to the mapping. The manuscript has been drafted by NA. GGP and MA have been involved in revision of the manuscript. All authors read and approved the final manuscript.
